# The Relationship between Dietary Protein Consumption and Risk of Fracture: a subgroup and dose-response meta-analysis of prospective cohort studies

**DOI:** 10.1038/srep09151

**Published:** 2015-03-16

**Authors:** Ai-Min Wu, Xiao-Lei Sun, Qing-Bo Lv, Yong Zhou, Dong-Dong Xia, Hua-Zi Xu, Qi-Shan Huang, Yong-Long Chi

**Affiliations:** 1 Department of Orthopaedics, Second Affiliated Hospital of Wenzhou Medical University, 109# XueYuan Xi Road. 325027, Wenzhou, Zhejiang, China; 2 Department of Orthopaedics, Tianjin hospital, 406 Jiefang Nan Road. 300211, Tianjin, China; 3 Department of Orthopaedics, Shanghai East Hospital, Tongji University School of Medicine, 150 Jimo Road. 200120Pudong, Shanghai, China

## Abstract

It is still debate of the relationship between the dietary protein consumption and risk of fracture. We searched Medline and Embase to assess the effects of dietary protein consumption on risk of fracture. Twelve prospective cohort studies with 407,104 participants were included, higher total protein consumption may be decrease 11% risk of hip fractures, with adj. RR of 0.89 (0.82, 0.97), no significant difference was found for total protein and risk of all fractures and limb fracture; for animal protein consumption and risk of all fractures and hip fracture, with adj.RR of 0.79 (032, 1.96) and 1.04 (0.70, 1.54); for vegetable protein consumption and risk of all fractures, hip fracture and limb fractures with adj.RR of 0.77 (0.52, 1.12), 1.00 (0.53, 1.91), and 0.94 (0.40, 2.22), the subgroup of vegetable protein consumption and risk of all fractures of postmenopausal women with adj.RR of 0.78(0.52,1.16). Dose-response meta-analysis the relationship of total/animal/vegetable protein and hip fracture was consistent to the results of forest plot, the line of total protein and hip fracture was below the Y = 1.0 line. This meta-analysis showed that total dietary protein consumption may be decrease the risk of hip fracture, but not for animal or vegetable protein.

Fracture is a significant cause of morbidity and mortality, especial for aged patients, and it is a challenging global burden^1^,^2^,^3^. One study predicted that may be the number of hip fractures will rise to about 6.26 million world widely at 2050^4^. How to prevent to fracture is a big issue among current researchers and doctors.

Protein is one of important factors that involved in bone metabolism. Beasley reported^5^ that the higher protein consumption could decrease the risk of hip and forearm fracture, and some other studies^6^,^7^ reported higher protein consumption was not associated with a decrease of fracture. Feskanich et al^8^ reported the high protein consumption may be increase the forearm fracture; overall, the reports were inconsistent.

Another problem is that may be different source of protein may be effect the risk of fracture. It was reported that animal protein might have a greater negative effect on bone health than vegetable protein^9^, because animal protein increase the urinary calcium excretion. However, the results were inconsistent to others studies^10^,^11^. Therefore, the relationship between protein consumption and risk of fracture was still debate.

The aim of this review is to evaluate the evidence from prospective studies on the relation between protein consumption and the risk of fracture, and to subgroup evaluate animal protein and vegetable protein consumption and the risk of fracture. To clear the risk of different site fracture, we evaluate the fracture by all fractures, hip fracture, vertebral fracture and limb fracture.

## Methods

The present study was accorded to the preferred reporting items for systematic review and meta-analyses (PRISMA) guidelines (Checklist S1)^12^.

### Search strategy

We searched the database of Medline and Embase on July 20, 2014, using the Key words of “dietary protein,” or “dietary animal protein,” or “dietary vegetable protein,” or “dietary plant protein” and “fracture,” or “hip fracture,” or “vertebral fracture,” or “limb fracture”. The function of “related article” was also used for search. The references of retrieved articles were manually searched to avoid initial miss.

### Selection criteria

Studies were included in this meta-analysis according to the following criteria: 1) designed as a prospective cohort study; 2) the exposure of interest in protein consumption or animal protein consumption or vegetable/plant protein consumption; 3) the primary outcome of interest in all fractures of the whole body or hip fracture or vertebral fracture or limb fracture; 4) the relative risk (RR) estimates with 95% confidence intervals (CI) were reported or could be calculated by data reported. If the data were duplicated and reported in more than one study, only the study of the largest number of cases was included. All potential studies were reviewed independently for eligibility by two authors (AMW and ZY), and any disagreement was discussed and resolved with the third independent author (XLS). If the data of dose, case of fracture and person-years could be extracted, it will be included into dose-response meta-analysis.

### Data extraction

Two reviewers (QSH and ZYH) independently extracted data for analysis, and the third reviewer checked the consistency between them. A standard data extracted form was used, including the first author's last name, publication year, sample size, country where the study was performed, the gender and age of participants, measure or exposure (Total protein or animal protein or vegetable protein), variables adjusted for analysis, and RR estimates with corresponding 95% CIs for each category of protein. If there were two or more RRs of different potential confounders, we extracted the RRs that reflected the greatest degree of control for potential confounders. If necessary, the primary authors were contacted to retrieve additional information. The study quality was assessed by using the Nine-Star Newcastle-Ottawa Scale^13^.

### Statistical analysis

The methods of statistical analysis of this study are refer to previous similar studies^14^,^15^. In STATA software, on the use of fixed effects model and random effects model for homogeneity data have the same results, therefore, we combine Study-specific RR using a random effects model, which considers both between study variation and within study^16^.

The protein was divided into three types: total protein, animal protein and vegetable protein; and the fractures were divided into four types: all fractures of whole body, hip fracture, vertebral fracture and limb fracture. If there were more than two studies report the same type protein and the risk of the same type fracture, the data will be pooled into meta-analysis, and the highest protein consumption category (Quartile or Tertile) *vs*. the lowest category were pooled for synthesis. If data of dose, case of fracture and person-years could be extracted from more than 2 studies about the relationship between one type protein consumption and risk of one type fracture, dose-response meta-analysis will be performed to analyze the relation of them. The method of dose-response meta-analysis was according to Orsini and colleagues, whereas the methods of random-effects meta regression models were according to Greenland and colleagues^17^,^18^.

In subgroup of vegetable protein consumption for the risk of all fractures of whole body, two studies^19^,^20^ reported the menopause status of women; therefore, we pooled them in another subgroup meta-analysis for postmenopausal women only.

Q and I^2^ statistics were used to evaluate the statistical heterogeneity^21^. Sensitivity analysis involved removing one study and evaluating whether the rest results would be markedly affected. Potential publication bias was evaluated by the method of Egger's regression asymmetry test^22^. All statistical tests were performed with the STATA software (version 12.0; StataCorp, College Station, TX, USA).

## Results

### Literature search

**Figure 1:**
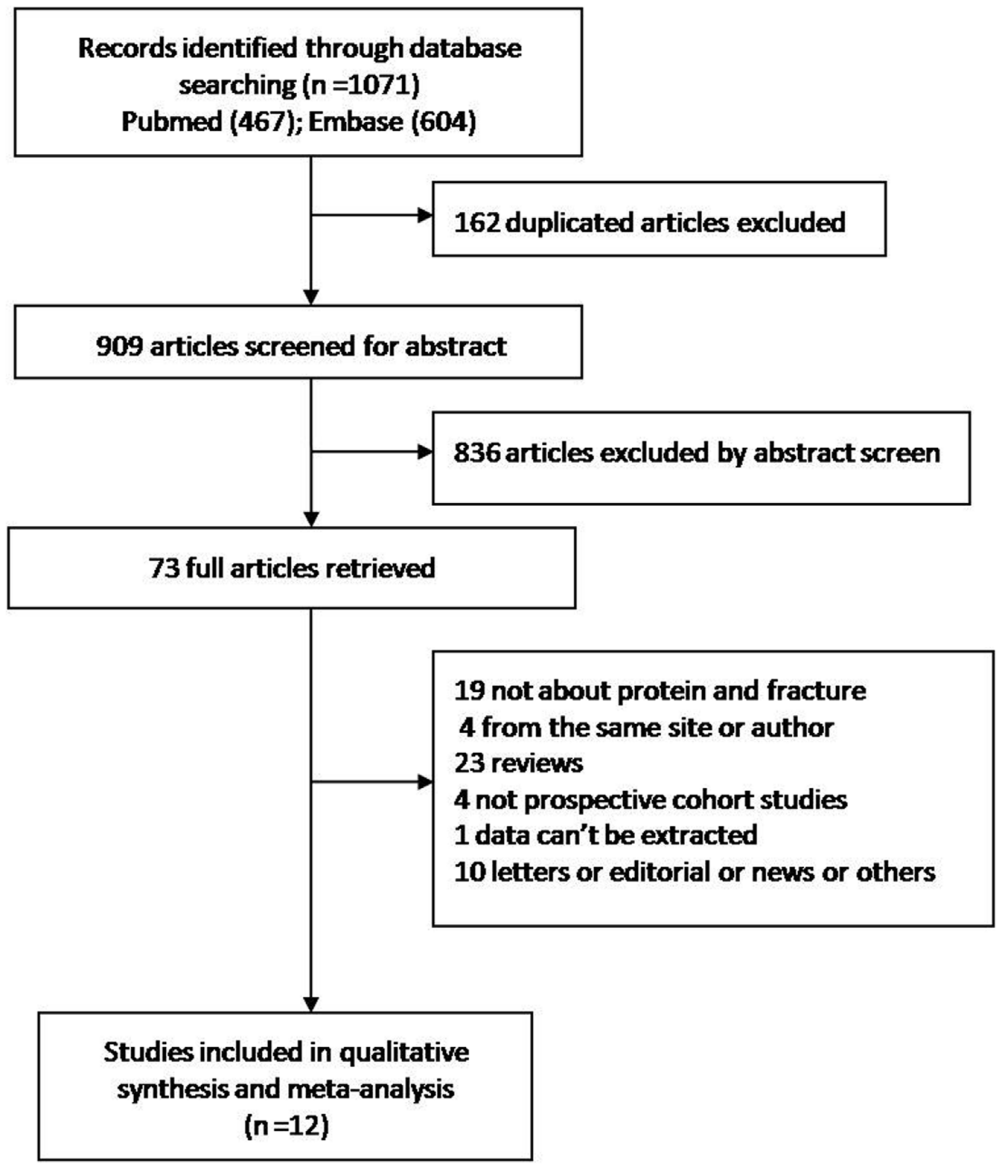
The selection of literatures for included studies.

The selection of literature for included studies is shown in Figure 1, total of 1071 potential records were identified from the databases, and 162 duplicated articles were excluded first, then the 836 articles was excluded by abstract screen, 73 full articles were retrieved, at last, 12 prospective cohort studies included for synthesis and meta analysis^5^,^6^,^8^,^11^,^19^,^20^,^23^,^24^,^25^,^26^,^27^,^28^ were included for meta analysis.

### Study characteristics

The characteristics of the included dietary protein consumption studies are showed in Table 1, and the dose of protein consumption of included studies were showed in Table 2. The total number of participants is 407,104 with 41,659 all fractures of the whole body, 4,000 hip fractures, and 9,599 limb fractures in the included 12 studies. Nine studies were conducted in the United States and Canada, two in Europe, and one in Asia. The Nine-Star Newcastle-Ottawa Scale results of included studies were showed in Table 3.

### Dietary total protein consumption and risk of fracture

**Figure 2:**
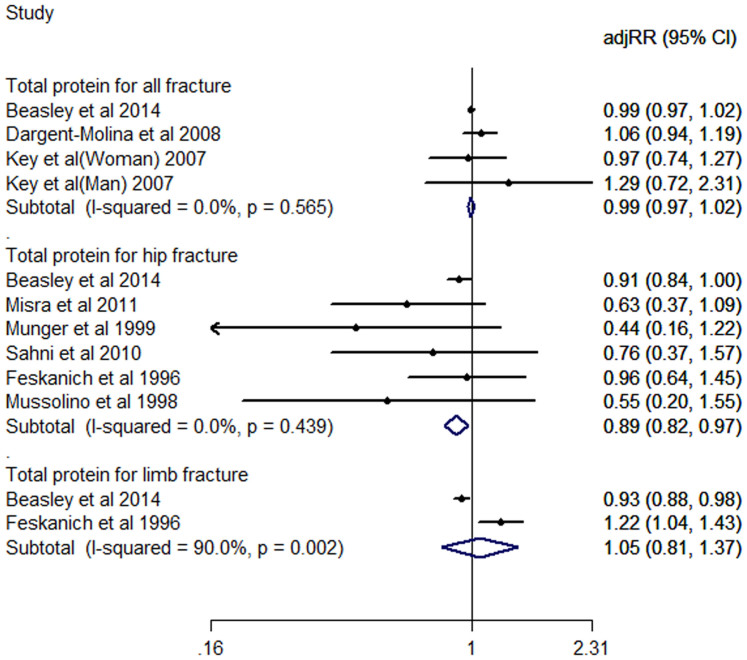
Adjusted Relative Risk of fracture (all or hip or limb fracture) for the highest vs. the lowest category of total dietary protein consumption.

Only one study^5^ concerns the relation between dietary total protein consumption and risk of vertebral fracture, therefore, cannot reach a meta-analysis. The total protein consumption and risk of all fractures of the whole body, hip fractures and limb fracture are shown in Figure 2. Our present meta-analysis of highest *vs*. lowest category shows that the adjusted relative risk (adj.RR) of total protein consumption for hip fractures is 0.89 (0.82, 0.97), has a statistic significantly difference, and decrease 11% risk of hip fractures. Others, for all fractures is 0.99 (0.97, 1.02), limb fractures is 1.05 (0.81, 1.37), no significant difference is found. Heterogeneity is observed at subgroup study of total protein consumption and risk of limb fractures (I^2^ = 90.0%, P = 0.002).

### Dietary animal protein consumption and risk of fracture

**Figure 3:**
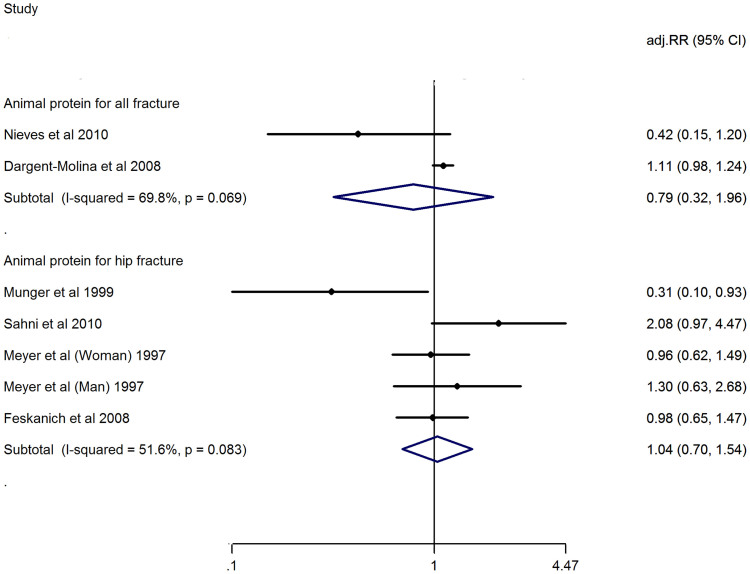
Adjusted Relative Risk of fracture (all or hip or limb fracture) for the highest vs. the lowest category of dietary animal protein consumption.

No study reported the dietary animal protein consumption and risk of vertebral fracture, only one study reported the dietary animal protein consumption and risk of limb fracture^8^, therefore, cannot reach meta-analysis of above two indications. The highest *vs*. lowest category shows that adj.RR of animal protein consumption for risk of all fractures of whole body is 0.79 (0.32, 1.96), and for risk of hip fractures is 1.04 (0.70, 1.54). Heterogeneity is observed at studies of risk of all fractures (I^2^ = 69.8%, P = 0.069), of hip fracture (I^2^ = 51.6%, P = 0.083). (Figure 3)

### Dietary vegetable protein consumption and risk of fracture

**Figure 4:**
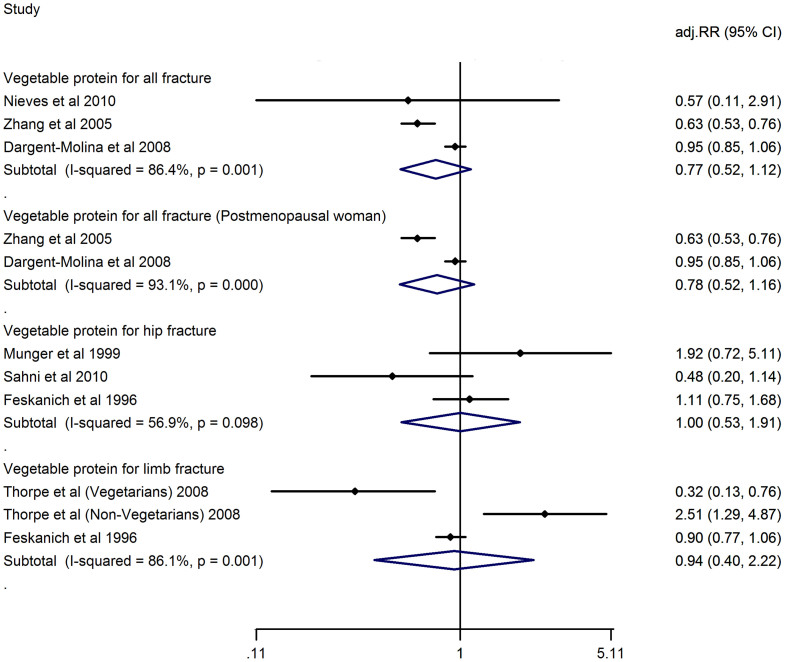
Adjusted Relative Risk of fracture (all or hip or limb fracture) for the highest vs. the lowest category of dietary vegetable protein consumption.

No study reported the dietary vegetable protein consumption and risk of vertebral fracture. The adj.RR of highest *vs*. lowest category of all fractures is 0.77 (0.52, 1.12), of hip fractures is 1.00 (0.53, 1.91), of limb fractures is 0.94 (0.40, 2.22). Heterogeneity is observed at studies of risk of all fractures (I^2^ = 86.4%, P = 0.001), of hip fracture (I^2^ = 56.9%, P = 0.098), of limb fracture (86.1%, P = 0.001). Two studies reported the vegetable protein consumption of postmenopausal women and risk of all fractures, the adj.RR of subgroup meta-analysis of postmenopausal women only is 0.78 (0.52, 1.16, I^2^ = 93.1%, P = 0.000). (Figure 4)

### Dose-response meta-analysis

**Figure 5:**
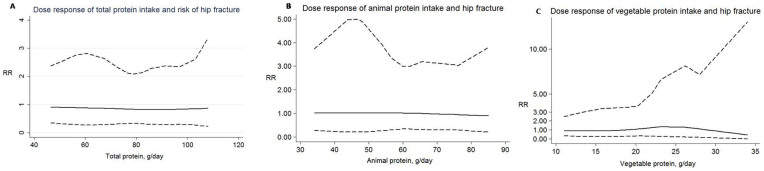
Dose-response relationship between total protein (A) or animal protein (B) or vegetable protein (C) and relative risk of hip fracture. Solid line represents adjusted relative risk and dotted lines represent the 95% confidence intervals for the fitted trend. The adj.RR of total protein intake and risk of hip fracture is below the line of RR = 1 (A), others two adj.RRs of animal protein intake and risk of hip fracture, vegetable protein intake and risk of hip fracture is spanning the line of RR = 1 (B and C). The result is consistent to the forest plot of Figure 2, 3 and 4.

Only the data of three sub-studies (total protein intake and risk of hip fracture, animal protein intake and risk of hip fracture, vegetable protein intake and risk of hip fracture) meet dose-response meta-analysis. The adj.RR of total protein intake and risk of hip fracture is below the line of RR = 1 (Figure 5A), others two adj.RRs of animal protein intake and risk of hip fracture, vegetable protein intake and risk of hip fracture is spanning the line of RR = 1 (Figure 5B/C). The result is consistent to the forest plot of Figure 2, 3 and 4.

### Sensitivity analysis and publication bias

The results of sensitivity analysis suggest that either the study of Dargent-Molina et al.^20^ or Zhang et al.^19^ omitted could decrease the heterogeneity of subgroup meta analysis of vegetable protein consumption and risk of all fractures, however, the studies of Dargent-Molina et al.^20^ or Zhang et al.^19^ both reported the risk of postmenopausal women, however, the study of Nieves et al.^27^ is not about postmenopausal women, therefore, combine study of Nieves et al.^27^ to either Dargent-Molina et al.^20^ or Zhang et al.^19^ is unreasonable. Therefore, we only did subgroup meta-analysis of postmenopausal women. The influence of each individual data set to the pooled RRs is not significant for all of other subgroup meta analysis (Supplemental Figures File 1). The Egger's test shows no evidence of publication bias of the total protein for all fractures or hip fracture (P = 0.286; P = 0.054), animal protein for hip fractures (P = 0.855), vegetable protein for all fractures or hip fractures or limb fractures (P = 0.701; P = 0.905; P = 0.949). Only two studies included in the subgroup meta-analysis of total protein for limb fractures and animal protein for all fractures, therefore, the Egger's test is error for them and the P value of Begg's test is P = 1.000 for both of them.

## Discussion

Fracture is a major global health problem. Many factors were supposed to decrease or increase the risk of fracture, such as age, BMD, physical activity, smoke, calcium, Vitamin D, Vitamin A and Vitamin K^29^,^30^,^31^,^32^,^33^. Protein is an important source of amino acid which to maintain bone structure, or stimulate some growth factors such as insulin-like growth factor I (IGF-I), then to increase the activity of osteoblast and the mineralization of bone matrix^34^,^35^, and the inadequate dietary protein may influence the bone strength and increase risk of fracture^36^,^37^. Some other concerns the relationship of high protein consumption and bone health are: 1) may be the protein will increase urinary calcium; 2) the protein could increase intestinal calcium absorption; 3) high dietary protein consumption may be act indirectly through preservation of muscle, and decrease falls and fractures^38^,^39^,^40^. However, the associate between the dietary protein consumption and risk of fracture is still dispute.

In 2009, Darling et al.^7^ meta analyzed the associate between the dietary protein and risk of hip fractures, three studies of total protein, three animal protein and two vegetable protein prospective reports were included by their study, and no associate between of dietary protein and risk of hip fracture was found at that time. In our present meta-analysis, added the recent publications, six studies of total protein, four of animal protein and three of vegetable protein prospective studies are pooled for analysis. We find that adjusted relative risk (adj.RR) of total protein consumption for hip fractures is 0.89 (0.82, 0.97), has a statistic significantly decrease 11% risk of hip fracture.

However, no benefit is found at meta-analysis of total protein for all fractures of the whole body and limb fracture. May be the included studies of them are still too small, only two or three different reports. Moreover, the hip fracture is more fragility than other sites, especially for aged participates; therefore, it may be prior fracture than other sites.

Some reports suggested that the different source of protein from animal or vegetable will be effect the risk of fracture varies. Sellmeyer et al.^9^ reported that more vegetable protein intake and less animal protein intake may decrease bone loss and the risk of hip fracture, however, Hannan et al.^41^ reported that higher animal protein consumption was not associated with a decrease in bone mineral density. In study of Munger et al.^10^, the higher animal protein intake had a lower risk of fracture than the lower animal protein intake category. In this meta-analysis, no difference is found by subgroup meta-analysis of animal protein and vegetable protein for all fractures, hip fracture and limb fracture. Because the higher total protein consumption is benefit for risk of hip fracture, may be this benefit is doesn't matter what the protein source from animal or vegetable.

The strength of our present meta-analysis study is that our quantitative assessment is based on prospective studies, compared to retrospective and case-control studies, these prospective studies minimizes the possibility of the recall and selection bias. In 2009, Darling et al.^7^ reported a meta-analysis of the associate between the dietary protein and risk of hip fracture; only four prospective studies were included for fracture risk meta-analysis at that time, without dose-response analysis. To the best of our knowledge, this is the first meta-analysis of the relationship between total/animal/vegetable protein and risk of all fractures, hip fracture and limb fracture based on prospective cohort studies, and a quantitative dose-response assessment of the relationship between protein consumption and risk of both hip fractures. Moreover, our study including the large number of participants, long duration of follow-up, and most individual studies are well powered.

There are also many limitations of our present study. Only one study report the risk of vertebral fracture^5^, therefore, cannot be meta-analysis. For some subgroup meta-analysis, such as total protein and limb fractures, animal protein and all fractures, the included studies are only two, and more prospective studies needed to be taken in future. Only the data of total/animal/vegetable protein and risk of hip fracture is sufficient for dose-response meta-analysis, others don't have enough data, and can't reach a dose-response meta-analysis.

Another limitation of this meta analysis is that: although the significant result data of total protein consumption for hip fracture without heterogeneity (I^2^ = 0.0%, P = 0.439), many other subgroup meta analysis have significantly heterogeneity, if these data have a significant result, which is suspected, the fortunate is that all of these heterogeneity data do not show any significant results.

## Conclusion

Total dietary protein consumption may be decrease the risk of hip fracture, but not for all fractures and limb fracture. No current evidence shows the animal or vegetable protein could decrease or increase the risk of fracture.

## Supplementary Material

Supplementary Information Supplementary file

Supplementary Information Checklist S1

Supplementary Information Dataset 1

Supplementary Information Dataset 2

Supplementary Information Dataset 3

Supplementary Information Dataset 4

Supplementary Information Dataset 5

Supplementary Information Dataset 6

## Figures and Tables

**Table 1:** Characteristics of Prospective Studies on Protein intake and Risk of Fracture

Author Year	No. of participants	Location/Period	Gender	Age (years)	No. of cases^a^	Measure/Exposure	Study Quality^b^	Adjustment for Covariates^c^
Mussolino et al. 1998	2,879	United States	M	45–74	HF: 79	Total protein	7	Smoking status, alcohol consumption, physical activity, chronic disease, calcium intake, calories, weight loss, bronchitis, thyroid disease, diabetes, kidney disease, coronary heart disease and stroke.
Beasley et al. 2014	144,580	United States	F	50–79	AF:36,166 HF:3,286 VF:4,836 LF:7,800	Total protein	7	Age, BMI, race-ethnicity, calibrated energy intake, general health, physical activity, history of fracture, history of parental fracture, smoking, corticosteroid, glucocorticoid use, treated diabetes, rheumatoid arthritis, and hormone use.
Meyer et al. 1997	39,787	Norway	F:19,752 M:20,035	35–49	HF:213	Animal Protein	7	Age, body height, BMI, serf-reported physical activity at work and during leisure time, diabetes mellitus, disability pension, marital status, and smoking.
Misra et al. 2011	946	United States	F:576 M:370	28–62	HF:100	Total Protein	7	Age, sex, weight, height and total energy intake.
Thorpe et al. 2007	1,865	United States and Canada	F	Post-menopausal or >45 Y	LF:171	Vegetable Protein	6	Education, BMI, practitioner-diagnosed medical conditions, coronary heart disease, stroke, high blood pressure, diabetes, diverticulitis, cancer, rheumatoid arthritis, other arthritis, alcohol use, smoking, nulliparity, menopausal status, age at menopause, hormone use and physical activity.
Nieves et al. 2010	125	United States	F	18–26	AF: 17	Animal protein Vegetable Protein	6	Baseline nutrient intake, beverage consumption, dietary patterns, treatment group assignment, Menstrual status, spine bone density, age, and fracture history.
Zhang et al. 2005	24,403	China	F	40–70	AF:1,170	Vegetable Protein	7	Age, BMI, hours of exercise, cigarette smoking, alcohol consumption, diabetes mellitus, education, family income, season of recruitment, calories intakes, calcium, fruits, and vegetables.
Munger et al. 1999	32,050	United States	F	55–69	HF: 44	Total Protein Animal Protein Vegetable Protein	7	Age, BMI, number of pregnancies, smoking, alcohol use, estrogen use, and physical activity.
Sahni et al. 2010	3,656	United States	F:1931 M:1725	26–86	HF: 44	Total Protein Animal Protein Vegetable Protein	7	Sex, menopause status, age, weight and height at baseline, physical activity index, intake of energy, vitamin D, smoking status, energy intake, dietary calcium intake.
Feskanich et al. 1996	85,900	United States	F	35–59	HF:234 LF:1,628	Total Protein Animal Protein Vegetable Protein	6	Questionnaire time period; Age; BMI, hours of activity; menopause status and HT use; cigarette smoking; use of thyroid hormone medication and thiazlde diuretics; alcohol and caffeine Intakes.
Dargent-Molina et al. 2008	36,217	France	F	40–65	AF: 2,408	Total Protein Animal Protein Vegetable Protein	7	BMI, physical activity, parity, maternal history of hip fracture, HT use, smoking status, and alcohol intake
Key et al. 2007	346,96	United Kingdom	F:26,749 M:7,947	20–89	AF:1,898	Total Protein	6	Age, smoking, intakes of energy and each other nutrient, alcohol consumption, BMI, walking, cycling, vigorous exercise, other exercise, physical activity at work, marital status and, for women, parity and HT use.

**Note**: a: AF: All fracture; HF: Hip fracture; VF: Vertebral fracture; LF: Limb fracture.

b: Study quality was judged based on the Newcastle-Ottawa Scale (range, 1–9 stars).

c: BMI: Body mass index; HT use: Hormone replacement therapy.

**Table 2:** The dose of different protein consumption of included studies

Study		Total protein	Animal protein	Vegetable Protein	Quartile
Mussolino et al. 1998 (g/day)	highest dose	>98	-	-	Q4
	lowest dose	<56	-	-	Q1
Beasley et al. 2014 (g/day)	highest dose	20% increased	-	-	
	lowest dose	-	-	-	
Meyer et al. 1997 (g/day)	highest dose	-	>20.6(Woman) >21.6 (Man)	-	Q4
	lowest dose	-	<13.6(Woman) <14.2(Man)	-	Q1
Misra et al. 2011 (g/day)	highest dose	82.74 ± 10.27	-	-	Q4
	lowest dose	46.45 ± 7.29	-	-	Q1
Thorpe et al. 2007 (Eight level food frequency)	highest dose	>1/day	-	-	Q3
	lowest dose	<3/week	-	-	Q1
Nieves et al. 2010 (g/day/kg)	highest dose	-	1 g/day/kg increased	1 g/day/kg increased	-
	lowest dose	-	-	-	-
Zhang et al. 2005 (g/day)	highest dose	-	-	>13.27	Q5
	lowest dose	-	-	<4.98	Q1
Munger et al. 1999 (g/day)	highest dose	>95.5	>75.14	>26.2	Q4
	lowest dose	<67.38	<43.74	<17.5	Q1
Sahni et al. 2010 (g/day)	highest dose	-	68	29	Q3
	lowest dose	-	38	18	Q1
Feskanich et al. 1996 (g/day)	highest dose	>95	>80	>19	Q5
	lowest dose	<68	<51	<12	Q1
Dargent-Molina et al. 2008 (g/1000 kcal)	highest dose	>50.11	>33.52	>14.12	Q4
	lowest dose	<40.75	<22.42	<10.07	Q1
Key et al. 2007 (g/day)	highest dose	>90	-	-	Q5
	lowest dose	<55	-	-	Q1

**Table 3:** Assessment of quality of included studies on the use of Nine-Star Newcastle-Ottawa Scale

	Selection					Outcome assessment			
Study (authors, year)	Representativeness of the exposed cohort	Selection of the nonexposed cohort	Ascertainment of exposure	Incident disease	Comparability	Assessment of outcome	Length of follow up	Adequacy of follow up	Score
Mussolino et al. 1998	*	*	-	-	**	*	*	*	*******
Beasley et al. 2014	*	*	-	*	**	-	*	*	*******
Meyer et al. 1997	*	*	-	*	*	*	*	*	*******
Misra et al. 2011	*	*	-	-	**	*	*	*	*******
Thorpe et al. 2007	*	*	-	-	**	-	*	*	******
Nieves et al. 2010	-	*	-	*	**	*	-	*	******
Zhang et al. 2005	*	*	*	-	**	-	*	*	*******
Munger et al. 1999	-	*	-	*	**	*	*	*	*******
Sahni et al. 2010	*	*	-	-	**	*	*	*	*******
Feskanich et al. 1996	-	*	-	-	**	*	*	*	******
Dargent-Molina et al. 2008	*	*	-	-	**	*	*	*	*******
Key et al. 2007	-	*	-	-	**	*	*	*	******

Note: One asterisk means one score, studies with more scores on behalf of higher quality.
