# Supplementary material for: The Relationship between Dietary Protein Consumption and Risk of Fracture: a subgroup and dose-response meta-analysis of prospective cohort studies
Source: Sci Rep. 2015 Mar 16;5:9151. doi: 10.1038/srep09151 (PMC5376209; doi:10.1038/srep09151)
Supplement: Supplementary Information — Supplementary file [file srep09151-s1.pdf]

Title: The Relationship between Dietary Protein Consumption and Risk of Fracture: A subgroup and dose-response meta-analysis of prospective cohort studies

Authors: Ai-Min Wu[1], Xiao-Lei Sun[2], Qing-Bo Lv[1]; Yong Zhou[1]; Dong-Dong Xia[3]; Hua- Zi Xu[1], Qi-Shan Huang[1*]; Yong-Long Chi[1].

Affiliated:

1. Department of Orthopaedics, Second Affiliated Hospital of Wenzhou Medical University, Wenzhou, Zhejiang, China.

Address: 109# XueYuan Xi Road, Wenzhou, Zhejiang, People's Republic of China

Postal code: 325027

2. Department of Orthopaedics, Tianjin hospital, Tianjin, China.

Address: 406 Jiefang Nan Road, Tianjin, China, 300211.

3. Department of Orthopaedics, Shanghai East Hospital, Tongji University School of Medicine, Shanghai, China

Address: 150 Jimo Road , Pudong, Shanghai, China, 200120.

Acknowledgement

This work is funded by National Natural Science Foundation of China (81372014, 81371988); and Xinmiao talent plan of Zhejiang Province (2014R413053). The funders had no role in the design, execution, and writing of the study.

Disclosures

All authors state that they have no conflicts of interest

Authors' roles:

AMW, QSH and YLC contributed to the conception and design of the study. AMW XLS DDX QBL HZX and YZ contributed to the analysis and interpretation of data. AMW YLC and QSH contributed to the drafting of the article. All authors revised the manuscript critically for important intellectual content and gave final approval of the version to be published. AMW and XLS are co-first authors on this study.

*** Correspondence to** Qi-Shan Huang MD Professor **E-mail:** hqshaq@126.com

Phone number: +86 0577 88002814

Fax number: +86 0577 88002823

Orthopaedic department, Second Affiliated Hospital of Wenzhou Medical University, Wenzhou, Zhejiang, China.

Address: 109# XueYuan Xi Road, Wenzhou, Zhejiang, People's Republic of China

Postal code: 325027

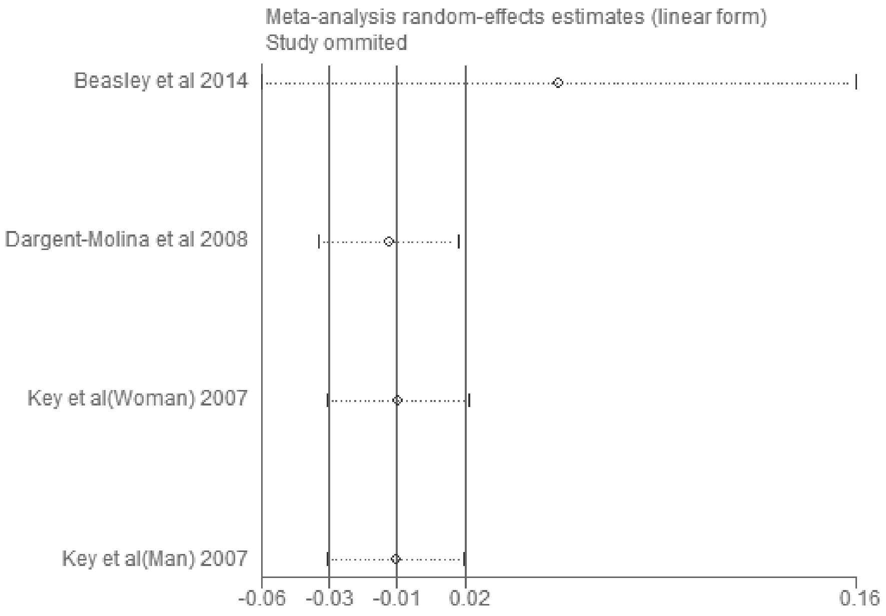

Figure S1: Sensitivity analysis of total protein and risk of all fractures.

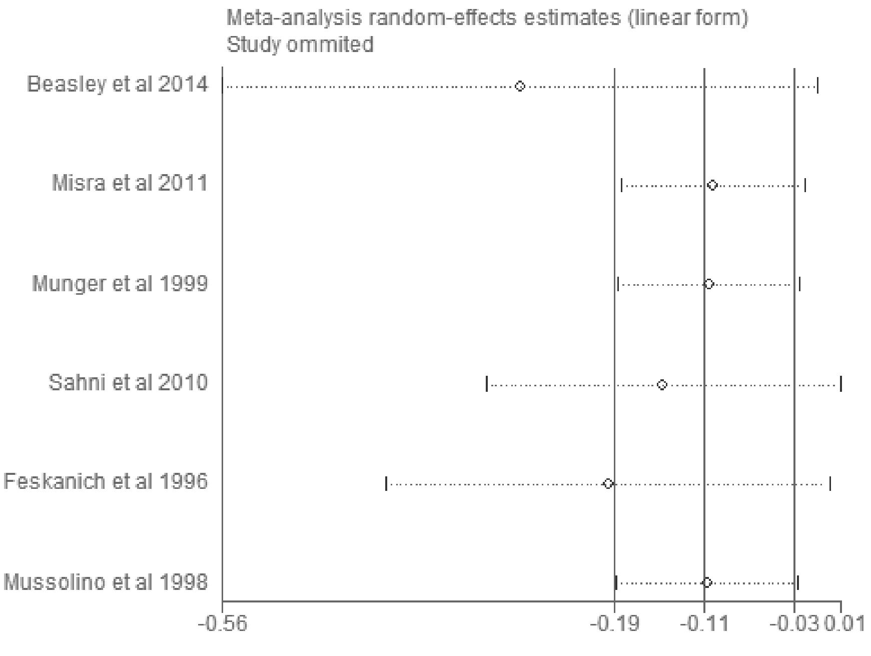

Figure S2: Sensitivity analysis of total protein and risk of hip fracture.

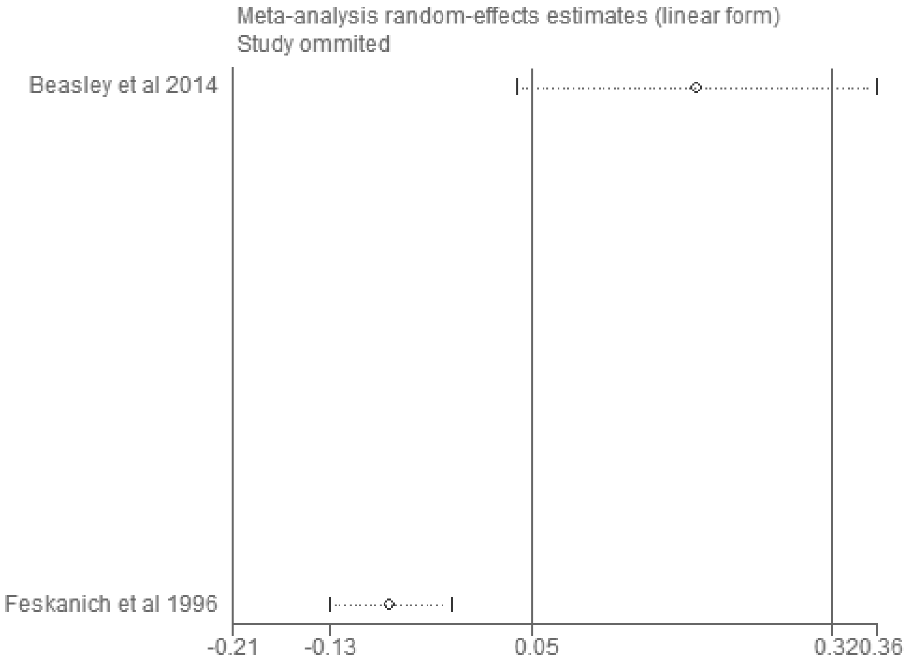

Figure S3: Sensitivity analysis of total protein and risk of limb fracture.

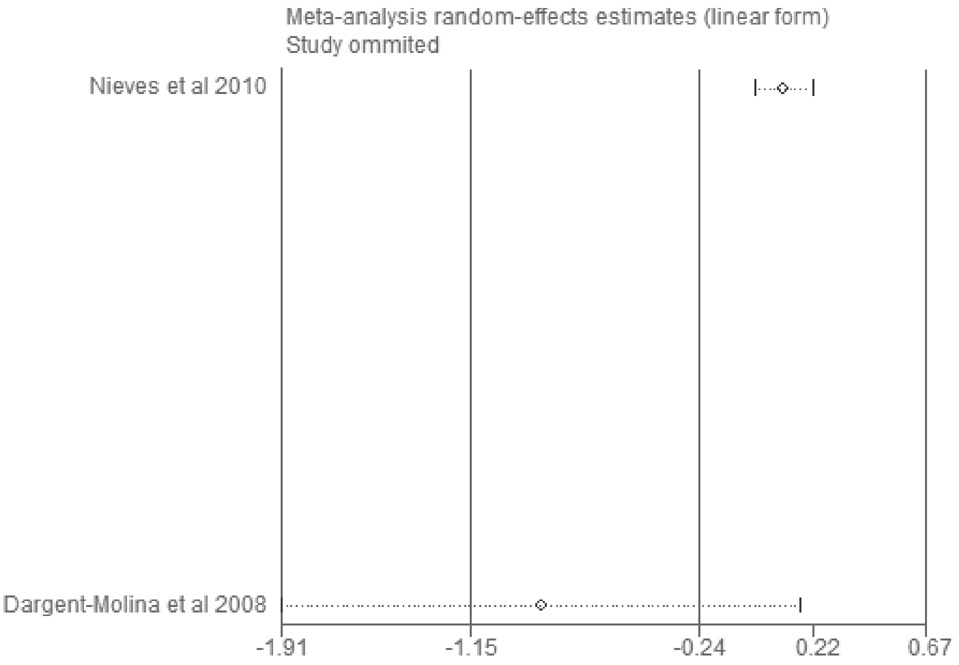

Figure S4: Sensitivity analysis of animal protein and risk of all fractures.

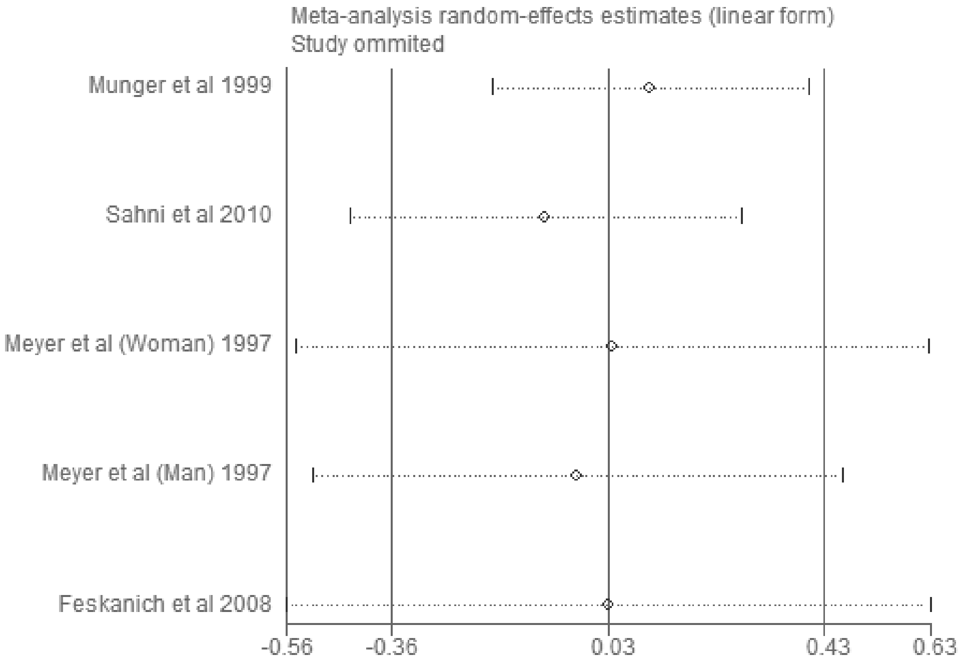

Figure S5: Sensitivity analysis of animal protein and risk of hip fracture.

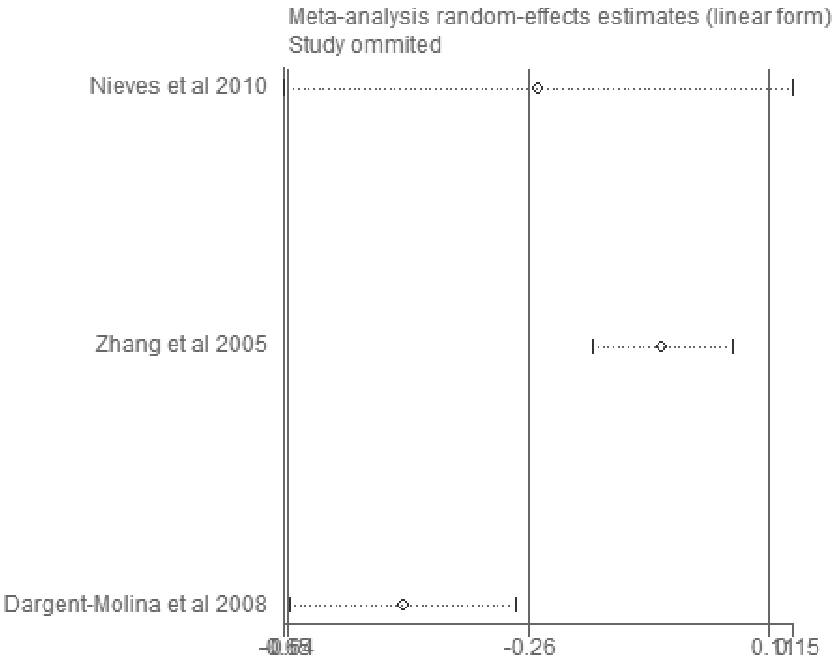

Figure S6: Sensitivity analysis of vegetable protein and risk of all fractures.

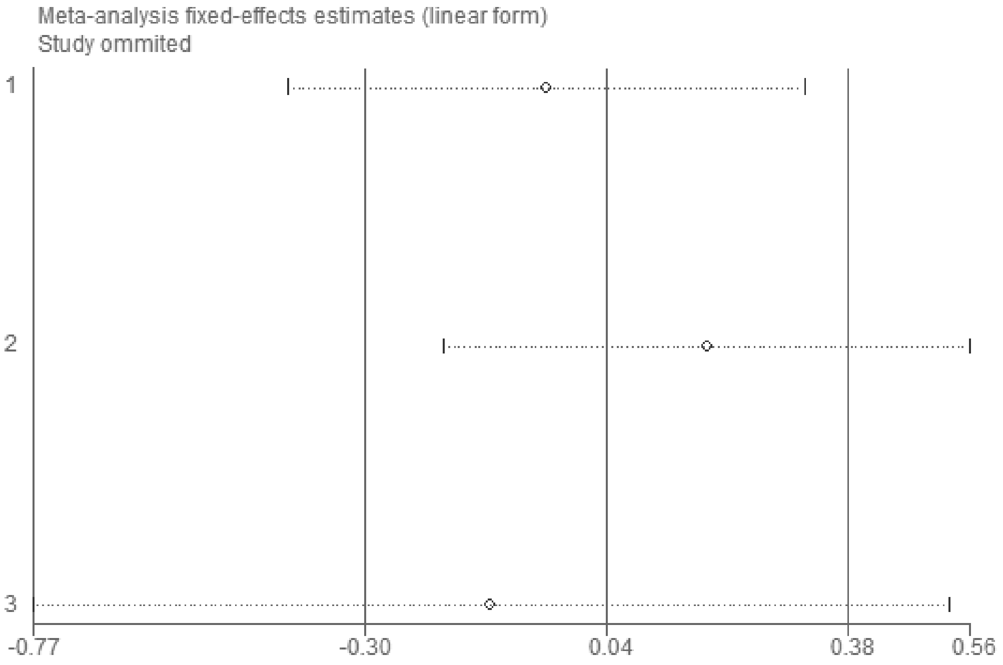

Figure S7: Sensitivity analysis of vegetable protein and risk of hip fracture.

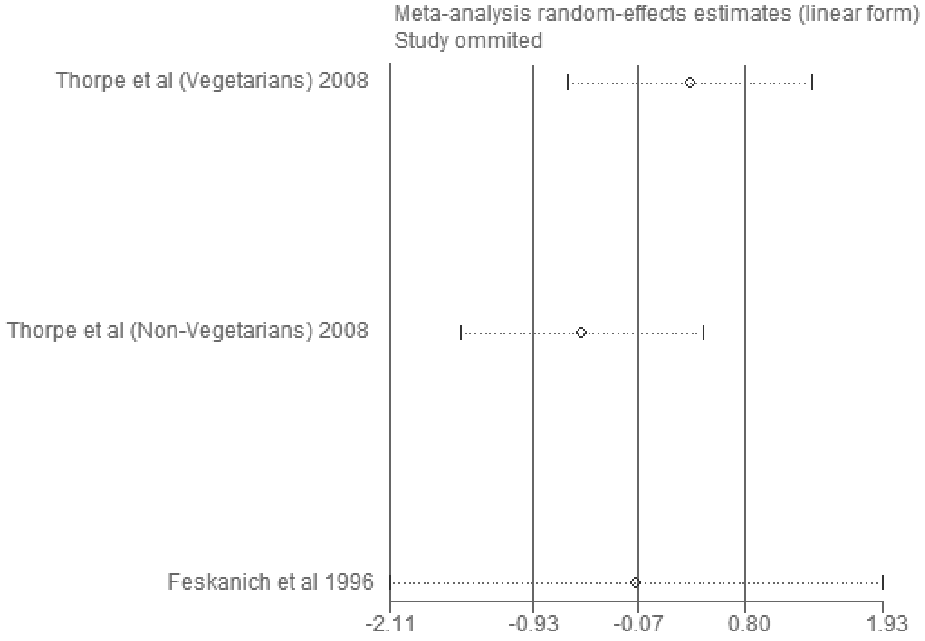

Figure S8: Sensitivity analysis of vegetable protein and risk of limb fracture.
